# Nuclear envelope morphology change upon repetitive treatment with modified antisense oligonucleotides targeting Hutchinson-Gilford Progeria Syndrome

**DOI:** 10.1016/j.bbrep.2022.101411

**Published:** 2022-12-28

**Authors:** Asmaa Abdelrahman, Mette-Marie Wendelboe Nielsen, Mette Halkjær Stage, Eva Christensen Arnspang

**Affiliations:** a Department of Green Technology, Faculty of Engineering, University of Southern Denmark, Odense, Denmark; b Department of Photochemistry, National Research Centre, Dokki, Giza, Egypt; c Department of Mechanical and Electrical Engineering, Faculty of Engineering University of Southern Denmark, Sønderborg, Denmark; d Department of Food Science, Faculty of Science, Copenhagen University, Copenhagen, Denmark

**Keywords:** Rare disease, Genetic mutation, Gene silencing, Confocal microscopy

## Abstract

We present the influence of treating progeroid fibroblasts with two modified antisense oligonucleotides (ONs) on the nuclear envelope. Two modified ONs were designed to block ribosome binding during translation and spliceosome binding at the cryptic splice site. We analysed the changes in the nuclear morphology of progeria cell nuclei after repetitive transfection with modified ONs as a physical analysis tool for estimating alteration of the gene expression at the protein level. Confocal microscopy was used to image the nuclei, and the nuclear lobulations were quantified to study the changes in the morphology of the nuclear envelope upon treatment. PCR was used to identify the changes in the expression of lamin A and progerin after antisense treatment at the RNA level. We found a significant decrease in the number of nuclear envelope lobulations and a lower progerin expression in progeria cells after transfection with modified ONs.

## Highlights

• Modified antisense strategy could help in silencing the gene expression responsible for progerin production.
• The regulation of the gene expression is determined via confocal microscopy of the cell nuclei and PCR analysis.
• Blocking the translation of the mRNA is more effective than blocking the activation of the cryptic splice site forming progerin.

## 1 Introduction

Hutchinson-Gilford Progeria Syndrome (progeria) is a rare pediatric premature ageing disease [1,2]. The symptoms for two years old children with progeria are weight loss, alopecia, joint contractures, and cardiac disease [2]. Progeria patients often die in their early teens with cardiovascular problems [3]. The reason for progeria is a de novo heterozygous mutation in the lamin A gene. This mutation is located in exon 11, codon 608, where a single base mutates from GGC to GGT. The mutation leads to an activated splice site and, thereby deletion of 150 nucleotides at the mRNA level. Subsequently, 50 amino acids are deleted at the C terminal end of lamin A, resulting in a truncated version of lamin A (progerin) [1]. The deleted region contains a cleavage site at the C terminus of prelamin A, and it is known that lamin A protein is one of the nuclear envelope components. Hence, the farnesylated carboxylated progerin is accumulated in the nuclear lamina instead of lamin A resulting in lobulations in the nuclear envelope in progeroid cells. This lobulated nucleus structure sterically hinders DNA copying during mitosis, increases DNA damage, and leads to rapid apoptosis of the cells. Hence, more progerin production would distort the nuclear membrane [3,4].

Some therapeutic approaches target progeria via inhibition of the production of progerin [1], for instance, the farnesyltransferase inhibitor lonafarnib prevents farnesylation of progerin C terminus [5,6]. Another treatment pathway is to use the mTOR inhibitor everolimus, which leads to the degradation of progerin, is in clinical trials right now [7]. Also, small molecules are used to reduce progerin levels, such as ICMT inhibitor C75 and Lonafarnib, which have been approved for use by FDA recently [[8], [9], [10], [11], [12], [13], [14], [15], [16], [17], [18]].

Antisense oligonucleotides (ONs) based therapeutic strategies have recently been described as promising approaches for treating various diseases. The therapy relies on blocking the expression of a target gene at the mRNA level via binding to a specific site of the mRNA [19,20]. Chemically modified nucleotides are critical for those treatment strategies aiming to enhance double helix stability, binding affinity, and nuclease resistance.

Antisense ON strategy has been used in some other diseases, including muscular dystrophy and spinal muscular atrophy [[19], [20], [21], [22], [23], [24], [25], [26]]. Therapeutically, antisense ON functions via RNA degradation by Rnase H attacking DNA–RNA hybrids formed between the target RNA and the ON or via sterically blocking [26].

For instance, morpholino-modified ONs were tested invitro, targeting the mutated splice site in lamin A, and antisense ON experiments performed in mice resulted in extended lifespan and inhibited the progerin's telomeric disorder [[27], [28], [29], [30]].

Many researchers have reported that the most effective antisense approach for progeria was targeting the splice site in exon 11 carrying the mutation site [27,29,31,32].

**Fig. 1:**
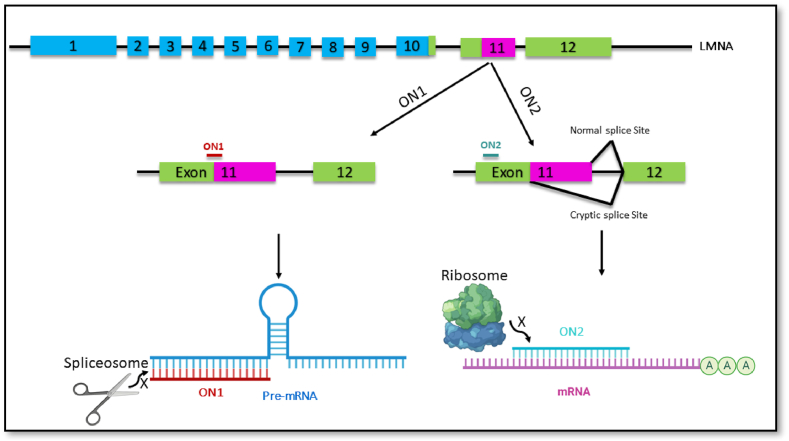
An overview of the binding sites of ON1 and ON2 in the LMNA gene; ON1: 5′-TCCACCCACCTGGGCTCCTC; ON2: 5′-GAGCGCAGGTTGTACTCAGC.

Hence, our in vitro study used the antisense strategy to silence a specific region in exon 11 via two strategies. We here present a repetitive in vitro transfection protocol for treating progeria using two modified antisense sequences targeting exon 11. The first antisense ON was designed to block the spliceosome from binding to the cryptic splice site [27]. In contrast, the second antisense ON was designed to hinder the ribosome via binding to mRNA sterically [27,33]. The two sequences have been modified with intercalating nucleic acid linker (INA) modifications designed to provide a stronger binding to the targeted DNA [34,35]. A repetitive transfection procedure was used to better study the effect of the ONs during the long-term delivery of the modified ONs. In (Fig. 1) is an overview of the effects of the antisense ONs.

We used two different human skin fibroblasts, one from a progeria patient and another from a healthy person. After the repetitive treatment, the cultures were fixed for imaging or harvested for PCR analysis. Confocal microscopy was used to image the nuclear membrane and study the nuclear morphology as a physical study for the production level of progerin upon antisense treatment. The lobulations per nucleus were counted on specific days. PCR was used to analyse the regulation of gene expression on the RNA level after the repetitive antisense transfection.

## 2 Results and discussion

We transfected progeria cells on the second, third and fifth days with INA-modified antisense ONs and unmodified ON as a control; in addition, healthy fibroblasts were cultured to be used as another control. Confocal microscopy was performed after staining cell nuclei with DAPI. A minimum of 50 nuclei for each sample was imaged and analysed (Fig. 3).

Quantification of the number of lobules per nuclei was performed manually and using ImageJ Particle8 [36,37]. The plugin evaluates circularity as a function of area and perimeter. The shape descriptor describes convexity as a function of the perimeter in the images. The convexity factor was tested, and a good tool for measuring the degree of lobulation was performed, and the results were comparable to the manual counts of lobulations.

**Fig. 2:**
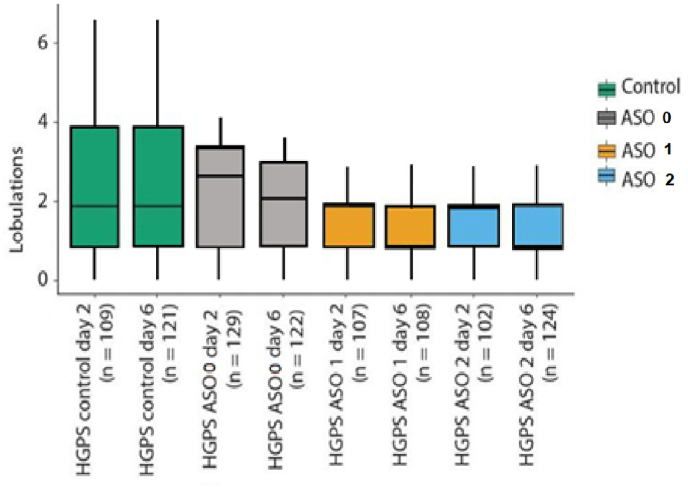
Manual count of lobulations of untreated progeria cells and treated cells with unmodified ON0 (controls), ON1 and ON2; ON1: 5′-TCCACCCACCTGGGCTCCTC; ON2: 5′-GAGCGCAGGTTGTACTCAGC.

Two cell lines were used for the experiments, a healthy control (GM01651) and one progeria cell line (AG03513); cells were seeded to a confluency of 70–90% and transfected with either unmodified oligonucleotide ON0, with the INA modified ones ON1 or ON2 every second day. DAPI staining was applied on the second-day, sixth-day and tenth-day samples. Samples were imaged by confocal microscopy, and the nucleus lobulations were manually counted twice and analysed with the ImageJ plugin. Any outliers were removed, and the dataset was examined with Welch's *t*-test (α = 0.05). In (Fig. 2) the results from the manual lobulation counts are shown.

**Fig. 3:**
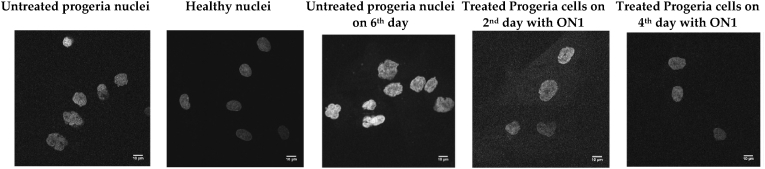
Examples of confocal microscopy images of untreated progeria nuclei, healthy nuclei (control), and treated cells with ON1; ON1: 5′-TCCACCCACCTGGGCTCCTC.

Both ONs decreased the number of lobulations in progeria cells. On the second day of the transfection, mean values of the lobulation were 2.670, 1.692, and 1.559 for control, ON1, and ON2, respectively, and treated samples of the last day of transfections (day 6), had mean values of 2.603, 1.426 and 1.371 of lobulation. Similar results were found using the convexity factor to evaluate the lobulation degree of the nuclei. Samples imaged after two days of treatment have means of 0.9340, 0.9422, and 0.9421, and cultures of the six days of treatment have mean values of 0.9320, 0.9465, and 0.9436.

**Fig. 4:**
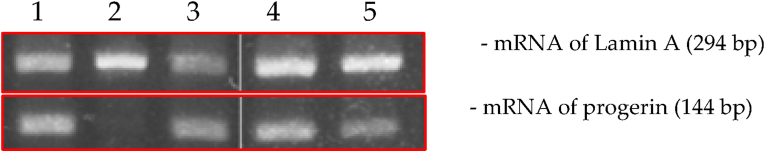
Agarose image of some of the PCR products, untreated progeroid cells on the first day (1st lane), untreated progeroid cells on the fifth day, untreated healthy fibroblasts (second lane), two times treated progeroid cells with ON1 (3rd lane) and two times treated progeroid cells with ON1 (4th lane) and four times treatment for progeria cells with ON1 (5th lane), respectively. ON1: 5′-TCCACCCACCTGGGCTCCTC.

On the other hand, other treated cultures were harvested for PCR analysis, and the PCR results of cell lysates of transfected cells show a decrease of the progerin expression on the 4th day with the treatment of progeria cells with ON1 (blocks binding of spliceosome) (Fig. 4).

Both confocal imaging and PCR analysis show a better result over repetitive transfection. In addition, the antisense oligonucleotide, designed to block the m-RNA of the cryptic splice site (ON1), provides a better down-regulation of progerin compared to the ON2, which is engineered to stop the premature RNA inside the nuclei. We successfully show that repeated transfection of antisense ONs containing intercalating nucleic acids is more efficient for treating progeria cells.

## 3 Materials and methods

### 3.1 Growth of the cells

The two utilised cell lines of human fibroblast cells for this experiment have been obtained from Coriell Cell Repository, USA. A control cell line from 13 years female with normal genotype, GM01651, in addition to 13 years male with progeria, AG03513.

Progeria cells were cultured in triplicates of 6-well plates VWR in a prewarmed medium 1:1 of (DMEM, 15% Fetal bovine serum (FBS), Gibco, United States, 1% penicillin and streptomycin solution(P/S), Corning, United States) and (MEM, 15% FBS, 1% Penicillin and streptomycin solution and 1 ml/100 ml non-essential amino acids (MEM NEAA), 100X, Gibco, United States) while the healthy cells were cultured in DMEM, 15% FBS, 1% P/S. The cells were incubated in a humidified atmosphere 5% CO2 incubator, Panasonic, Japan, at 37 °C and checked with the light microscope, Motic, AE31E, United States, until they reached 90-70% confluent.

### 3.2 Repetitive transfection

The fibroblasts were treated with two INA-modified ONs from Pentabase ApS, Denmark. The primary sequences are ON1: 5′- TCCACCCACCTGGGCTCCTC; ON2: 5′- GAGCGCAGGTTGTACTCAGC. Also, as a further control, both cell lines were grown without transfection.

The growth media was changed 1 h before the transfection of the cells of 70% confluent. The transfecting solution was prepared via dilution of 1:25 (v/v) lipofectamine™3000, Invitrogen™, United States in Opti-MEM™ reduced serum medium, Gibco, United States, then vortex 2–3 s. Also, ON mixtures containing 5.5 nM of ON in Opti-MEM medium were prepared. Then, 1:1 of the ONs mixture and the diluted Lipofectamine 3000 reagent were mixed in a tube and incubated for 15 min at room temperature. Afterwards, the prepared lipid complex was added to the cells. The transfection step was repeated on the experiment's 3rd, 5th, 7th, and 9th days. The cultures were split on the 4th and 8th days to avoid stressing the cells.

### 3.3 DAPI staining

The treated and untreated cultures were seeded on coverslips in 6 well plates. Then they were washed with warm PBS to be fixed with 4% formaldehyde, Sigma-Aldrich, United Kingdom. After one day of each transfection, the cells were stained for imaging, using 0.1% Triton X-100 solution and 300 nm DAPI, Sigma-Aldrich. Then the cells were stored in PBS at 5 °C until confocal imaging.

### 3.4 Confocal microscopy

The DAPI stained nuclei were imaged using a Zeizz LSM 510 Meta microscope with a two-photon Spectra-Physics Mai-Tai laser and a Plan-Apochromat 63x/1.4 Oil DIC objective. Also, the used laser is set up at 740 nm, maximal pinhole size, frame size was set to 1024 × 1024 pixels, and scan time was 2 min and 5 s, corresponding to a pixel dwell of 25.60 μs.

### 3.5 Nuclei lobulation counting

Images were analysed in ImageJ. First, the maximum intensity projection was used, and the nuclei were identified via threshold adjustment. The lobulations for each nucleus were then counted.

### 3.6 Gene expression

Both cell lines were grown in triplicates; afterwards, the RNA was purified, and cDNA was synthesised using reverse transcriptase. Finally, the targeted gene expression regulation was studied using polymerase chain reaction and confocal microscopy, as described below [38].

### 3.7 RNA purification

The cultures were harvested using the standard protocol by QIAzol Lysis Reagent, QIAGEN, Germany.

Harvested cells were treated with 0.2 (v/v) chloroform and then vortexed for 30 s. Then the mixture was incubated for 15 min at room temperature and spun for 10 min at 10.000 g at 4 °C. The upper phase was transferred to a 1.5 ml tube and placed on ice. Also, 0.6 (v/v) of 96% ethanol Sigma-Aldrich, United Kingdom, was added briefly, and the mixture was vortexed, then the eluent was loaded to an EconoSpin column, Epoch Life Science, United States, and spun at 13.000 g for 30 s at room temperature. The column was washed three times with 500 μl of buffer RPE, QIAGEN, Germany, at room temperature at 13.000 g for 30 s. The column was dried by spinning at 13.000 g for 2 min at room temperature. The column was transferred to a new 1.5 ml tube and left to further dryness for 5–10 min at room temperature with the lid open.

Then the purified RNA was eluted out of the column with 75 μl DEPC- treated water and incubated for 2 min at room temperature. After that, the column was spun at 16.000 g for 1 min. Finally, the concentration and quality of the RNA were determined by Nanodrop ND-100 Spectrophotometer, Thermo Fisher, United States, at 260/230 nm.

### 3.8 Reverse transcription

cDNA was synthesised in a 1.5 ml tube via mixing 0.3 μg of purified RNA, 2 μl 5X DNase buffer (1 μl 5X 1st Strand Buffer), Invitrogen™, United States, and 1 μl 10 DNaseǀ, Invitrogen™, United States, and DEPC water to the final volume of 10 μl, then gently the mixture was shacked and incubated for 15 min at 37 °C. The tube was placed on ice, and 3 μg of random hexamers, Invitrogen™, United States, were added. Subsequently, the sample was vortexed and then spun down briefly. Later, the samples were incubated for 5 min at 85 °C and then rapidly placed on ice.

For the reverse transcription, a mixture of 5 μl 1st strand buffer, 2.5 μl of 100 mM dichlorodiphenyltrichloroethane (DTT), Invitrogen™, United States, 1 μl DEPC water, 2.5 μl of 10 mM of dNTP mix, Thermo Fisher, United States, and 1 μl of Moloney murine leukaemia virus (M-MLV), Invitrogen™, United States, were prepared.

The reverse transcription mix was added to the sample, kept at room temperature for 10 min, then incubated for 1 h at 37 °C followed by adding 290 μl ultra-pure water to the reaction and stored at −80 °C.

### 3.9 Polymerase chain reaction (PCR)

PCR reactions were performed using 10 μM forward primer (5′-GGCTCCCACTGCAGCAGCTC) and 10 μM reverse primer (5′-CATGATGCTGCAGTTCTGGGG), Pentabase ApS, Denmark. The reagents were thawed and centrifuged before use. A PCR master mix was prepared by mixing 1X of 10X PCR buffer without magnesium, Invitrogen™, United States, 1.5 μM of 1X, 50 mM MgCl_2_, Invitrogen™, United States, 0.2 mM of 10 mM dNTP mix, Thermo Fisher, United States, 1U/rxn Taq DNA polymerase (5 U/μl), Invitrogen™, United States, and water to final volume 25 μl. Then the PCR master mix was mixed and briefly centrifuged. 0.5 μM of reverse, forward primers and cDNA were added to the master mix. Then the tube was capped, and the contents were centrifuged rapidly. The reaction was incubated in the thermal cycler T100-thermal cyclers, Bio-rad, Denmark, using initial denaturation at 94 °C for 3 min, 40 cycles consisting of denaturing at 94 °C for 45 s, annealing at 58 °C for 30 s, extension at 72 °C for 90 s. The final extension was performed at 72 °C for 10 min, and the hold was set at 4 °C.

### 3.10 Gel electrophoresis

PCR products were visualised by gel electrophoresis using 3% agarose running at 100 V for 1.5 h in Tris-acetate-EDTA (TAE) buffer consisting of 40 mM 2-amino-2-(hydroxymethyl)-1,3-propanediol (Tris), Sigma-Aldrich, 20 nM acetic acid, Sigma-Aldrich, and 1 mM Ethylenediaminetetraacetic acid (EDTA), Sigma-Aldrich. The agarose was stained with 1X SYBR safe DNA gel stain, Thermofisher, and a low-range ladder; Invitrogen was used. The gel was visualised on ChemiDoc XRS + Biorad.

## 4 Conclusions

This in vitro study employed a repetitive delivery method. Two cell lines, healthy and progeroid skin fibroblasts, were transfected using two modified INA-ASOs every second day for ten days. In addition, both progeroid fibroblasts which were treated with unmodified ASO, and the untreated cells were used as a control. The two modified ASOs were chosen either to block the production of the mRNA of the progerin or the translation of the mRNA into protein. The regulation of the gene expression at the RNA level was measured via PCR analysis.

Furthermore, the production of the undesired protein (progerin) was analysed using confocal microscopy to image the cell nuclei and then quantify the changes in the nuclear morphology of the membrane upon the repetitive INA-modified ASO treatment. The quantitative analysis of the degree of the nucleus lobulations using fluorescence microscopy was an effective tool to investigate the gene expression at the protein level of the progerin in the treated cells. A noticeable decrease was observed in the number of lobulated nuclei after three times the ASO transfection, which agrees with the PCR results. We conclude that the repetitive transfection technique was effective in downregulating progerin. Also, we found that blocking the translation of the mRNA more efficiently lowered the mRNA expression of progerin and the lobulation degree in nuclei than blocking the binding of the spliceosome to the cryptic splice site in the tested skin progeroid cells.

## Funding

“Villum Foundation funded this research, Grant number 19105 to E. A. C., and 10.13039/501100002808Carlsberg Foundation Grant number CF14-0786 to E. A. C.”

## Author contributions

“Conceptualization and supervision, E. A. C.; methodology, analysis, and investigation, M. W. M.; analysis and investigation M. H. S.; analysis, writing, reviewing and creating graphs, A. A.; funding acquisition, EAC All authors have read and agreed to the published version of the manuscript.”

## Data access statements

“All data supporting this study are provided in full in the 'Results' section of this paper”.

## Declaration of competing interest

“The authors declare no conflict of interest. Also, the funders had no role in the design of the study; in the collection, analyses, or interpretation of data; in the writing of the manuscript, or in the decision to publish the results”.

## Data Availability

No data was used for the research described in the article.
